# Effectiveness of a Multidisciplinary Limb Preservation Program in Reducing Regional Hospitalization Rates for Patients With Diabetes-Related Foot Complications

**DOI:** 10.1177/15347346241238458

**Published:** 2024-03-20

**Authors:** Ali Manji, Reza Basiri, Francois Harton, Kenton Rommens, Karim Manji

**Affiliations:** 1 Zivot Limb Preservation Centre, Alberta Health Services, Calgary, Alberta, Canada; 2 Department of Surgery, 70401 Cumming School of Medicine, University of Calgary, Calgary, Alberta, Canada; 3 Institute of Biomedical Engineering, University of Toronto, Ontario, Canada

**Keywords:** hospitalization, diabetic foot complications, ulcers, peripheral arterial disease

## Abstract

**Objective:**

This study evaluated the toe and flow model (TFM), a limb preservation program led by podiatric surgeons in Alberta, Canada, for its impact on hospitalization rates and length of stay (LOS) in patients with diabetic foot complication (DFC). Diabetes, a leading cause of non-traumatic lower extremity amputations (LEAs) in Canada, often results in diabetic foot ulcers (DFUs), a major cause of infection, amputation, and hospitalization. TFM has reportedly reduced amputation rates by 39% to 56%.

**Methods:**

The study analyzed Alberta's health database from 2007 to 2017, focusing on diabetes patients aged 20 and above. It included patients with various DFCs and compared outcomes in regions using TFM and standard of care (SOC). The study also examined data from two major cities, one with TFM and the other without, including rural referrals to Calgary and Edmonton. The data were normalized for the diabetic population and analyzed using a standard Student’s *t*-test.

**Results:**

TFM regions showed significantly lower hospitalization rates (*p* = 1.22E-12) than SOC regions. Over 11 years, TFM maintained lower average and median LOS by 0.13 and 0.26 days, respectively. TFM access reduced hospitalization risk by up to 30%, and patients in TFM regions had a 21% shorter LOS compared to SOC regions.

**Conclusion:**

Despite similar demographics and healthcare systems, the TFM region benefited from a dedicated multidisciplinary program and comprehensive limb preservation services. The study shows that TFM effectively reduces hospitalizations and LOS for DFCs, with significantly better outcomes in the TFM region than in SOC regions.

## Introduction

In Canada, diabetes is the primary cause of non-traumatic lower extremity amputations (LEAs), with around 70% of amputations necessitating hospitalization.^1–3^ Patients with diabetes who have peripheral neuropathy and peripheral vascular disease face an elevated risk of developing diabetic foot ulcers (DFUs), with a prevalence of 25% among individuals with diabetes.^5–7^ DFUs are the leading cause of infection, amputation, and hospitalization in patients with diabetes.^8^ From 2005 to 2016, some major medical centers experienced an overall increase in LEA rates in individuals over 40 years old, primarily due to diabetes, peripheral arterial disease, or a combination of both.^4^

To prevent LEA-DFUs, early identification and regular screening of risk factors such as peripheral neuropathy, peripheral arterial diseases, and foot deformities are crucial. The toe and flow model (TFM), a podiatry-led multidisciplinary approach, emphasizes coordinated care and aids in the early detection of these risk factors, resulting in reduced LEA rates.^4,9^ Implementing TFM or similar models in Europe, the United States, and Canada has shown a potential reduction in amputation rates by approximately 39% to 56%.^9,10,12^

The TFM model involves collaboration among dedicated and specialized disciplines to achieve optimal patient outcomes.^9,11^ While existing literature supports the decrease in LEA rates with the TFM approach, limited longitudinal data is available on its impact on hospitalization rates and length of stay (LOS). Given that roughly 70% of amputations in Canada require hospitalization, along with other diabetes-related foot complications (DFCs) that may not lead to amputation but often necessitate admission, we aimed to investigate hospitalization rates and LOS in regions with and without TFM systems.^3,9,10,12^ The innovative aspect of this study lies in examining the impact of TFM on hospitalization and LOS within the unique, large-scale, and enclosed setting of Alberta. This region operates under a cohesive provincial universal health care system and shares similar demographic characteristics, with the notable distinction that the Calgary area benefits from a well-established TFM center. In Calgary, Alberta, the TFM model is founded on a synergistic collaboration between the Podiatric Surgery and Vascular Surgery sections at the University of Calgary. This integration is exemplified by the Podiatric Surgery Service, which offers consultative and surgical expertise through the Zivot Limb Preservation Centre, emergency departments, and inpatient hospital services. As a publicly funded entity, Podiatric Surgery is a comprehensive service offering elective, urgent, and emergency surgical interventions. It specializes in limb preservation procedures, primarily addressing complications arising from diabetes, and serves approximately 2 million people. The Podiatric Surgeons within the Calgary TFM framework function as primary coordinators for limb preservation services, encompassing initiating treatment, surgical intervention, and orchestrating multidisciplinary consultations. This collaborative approach involves specialists from vascular surgery, plastic surgery, orthopedics, internal medicine, infectious diseases, endocrinology, and pedorthics, ensuring a holistic treatment paradigm for patients.

This study aimed to compare hospitalization rates and LOS associated with DFC in two regions of Alberta, Canada. In the Calgary region of the province, an active TFM was established in 1998, with subsequent expansions in 2006 and 2016.^12^ For comparison purposes, we examined a similar regional zone that followed the standard of care (SOC) and lacked a formal multidisciplinary limb preservation program.

## Methods

**Figure 1.**
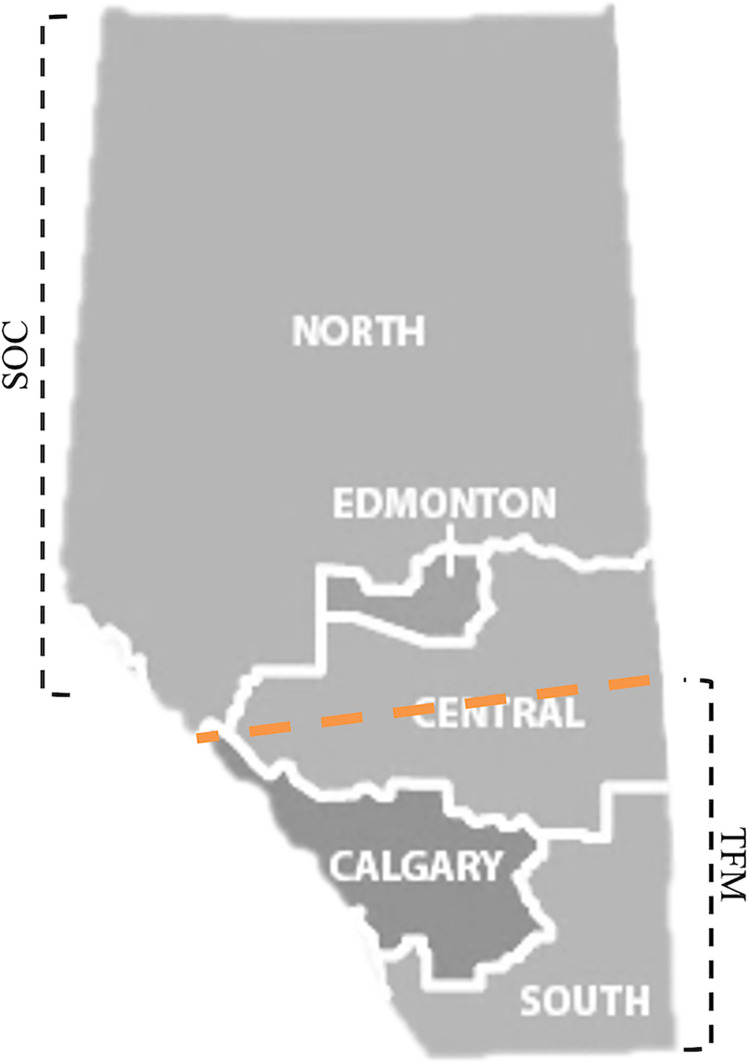
Provincial health region divisions according to Alberta Health Services and our proposed SOC and TFM regions. We investigate hospitalization and LOS values in the standard five regions and our adopted two-region approaches. In the two-region approach, geographically neighboring North and South regions are grouped.

We searched the province health database for patients with records of confirmed diabetes who were 20 years of age or older at the time of admission from 2007 to 2017. The study included patients who were hospitalized with various conditions related to diabetic foot complications, including diabetic ulcers of the lower limbs (such as decubitus/pressure ulcers, skin ulcers, varicose veins with ulcers), gangrene, atherosclerosis of extremities with gangrene, cellulitis of lower limbs, and osteomyelitis of lower limbs. The year 2007 marked the earliest available data from the Alberta provincial data center. Institutional review board approval was waived as this investigation was deemed a quality improvement project. The results were then classified into five standard geographical regions defined by Alberta Health Services: (1) Calgary, (2) Central, (3) Edmonton, (4) North, and (5) South, as depicted in Figure 1.

To account for referrals from rural areas to the two major tertiary care sites, Calgary and Edmonton, we also analyzed data from two central metropolitan regions: one with a limb preservation program (TFM region) and one without (SOC region). The TFM region encompassed Calgary, the South zone, and the southern half of the Central zone, while the SOC region comprised Edmonton, the North zone, and the northern half of the Central zone.

To assess hospitalization rates in the province, we gathered information on the number of hospitalizations (with a minimum one-night stay) and calculated the means and median LOS for each year. We normalized the hospitalization rates by dividing the values for each region and year by the corresponding historical diabetes patient population in that zone. Similarly, for LOS analysis, we employed a normalization approach based on the diabetes patient population of each region. The total LOS was determined by multiplying the mean LOS by the normalized hospitalization rates. This method allowed us to investigate the average number of days a diabetes patient spends in hospitals within each region. We performed hospitalization rate and total LOS calculations twice: once for the original five regions and again for the adopted TFM and SOC regions. To evaluate statistical significance, we utilized the standard Student’s *t*-test (*p* < 0.05) for the 11-year data set of normalized hospitalization rates and total mean LOS values among the regions.

## Results

### Hospitalization

**Figure 2.**
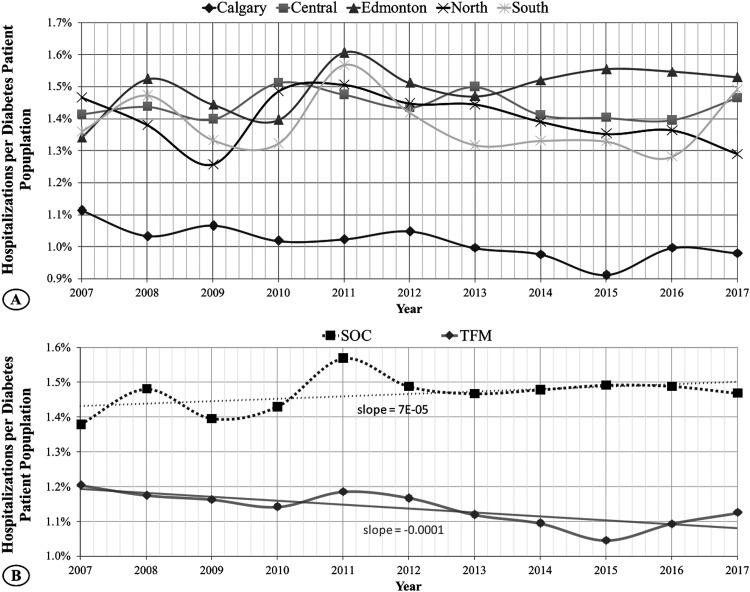
Eleven years of normalized hospitalizations rates. (A) Calgary region with TFM maintains the lowest and decreasing number of hospitalizations per diabetes patient population over this time. (B) In the two-region comparison, TFM, containing Calgary and immediate surrounding zones, continues to demonstrate lower and decreasing hospitalization values compared to the SOC region that includes Edmonton and immediate surrounding zones. The risk of hospitalization is decreased by up to 30% in TFM-accessible regions.

In the initial analysis of the standard five regions, it was observed that Calgary had the lowest number of hospitalizations per diabetes patient population, as depicted in Figure 2A. On average, approximately 1.0 ± 0.05% of diabetes patients in Calgary were admitted to the hospital over 11 years. The rates in the other four regions were significantly different from Calgary (*p*-values < 3.0E-09), but their hospitalization rates within each other were similar, ranging from 1.4% to 1.5% ± 0.07%. The average rates over the 11 years for the other four regions were as follows: Central: 1.44 ± 0.04%, Edmonton: 1.50 ± 0.07%, North: 1.40 ± 0.08%, and South: 1.38 ± 0.09%.

Even considering the larger SOC and TFM regions, the TFM group demonstrated a significantly lower normalized hospitalization rate (*p* = 1.22E-12) than SOC, as illustrated in Figure 2B. On average, over 11 years, the SOC zone had 1900 ± 392 hospitalizations, while the TFM zone had 1338 ± 241 hospitalizations. This corresponds to approximately 1.5% and 1.1% hospitalizations per diabetes patient in the TFM and SOC regions, respectively. The hospitalization rate in the SOC group displayed a slightly increasing trend over the 11 years, whereas, in the TFM group, this rate decreased over time. When considering the individual regions, there was a reduction in the risk of hospitalization by up to 30% in TFM-accessible regions.

### Length of Stay

**Figure 3.**
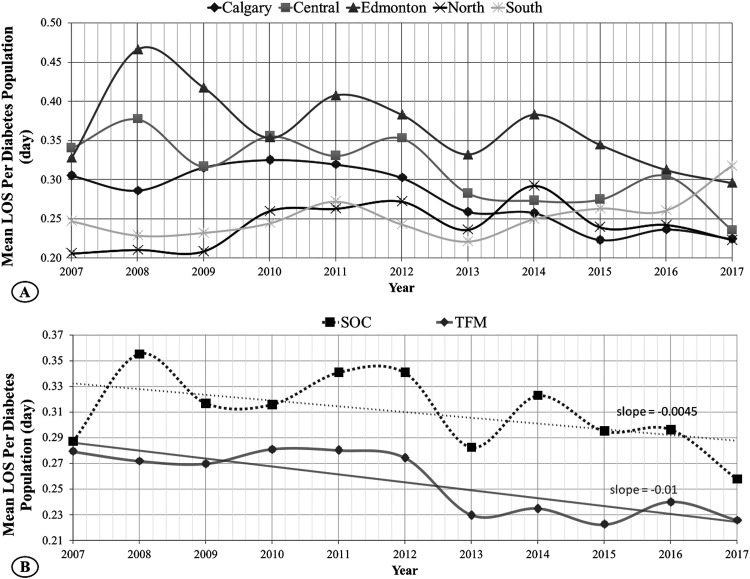
Eleven years of normalized mean LOS. Mean LOS indicates the total average number of days spent in a hospital per individual diabetes patient. (A) TFM hub in Calgary is experiencing a significantly lower LOS than the SOC hub in Edmonton. The TFM group shows a faster decreasing LOS rate. (B) In the two-group comparison, while both TFM and SOC regions demonstrate decreasing LOS, the TFM region maintains, on average, 21% less LOS when compared with SOC.

As shown in Figure 3A, Edmonton had the highest overall mean and median for total LOS of 0.37 and 0.20, respectively, over the 11 years. The North region had the lowest overall mean and median for total LOS of 0.24 and 0.11, respectively. The remaining three regions had an average median LOS of 0.14. While the median trends for every region pair were significant, we found no significant difference between Calgary and Central, Calgary and South, and Central and South.

In the two-region approach (Figure 3B), the cumulative TFM region maintained a lower mean and median for LOS of 0.13 and 0.26 days, respectively. In comparison, SOC had 0.15 and 0.31 days for mean and median LOS values. The mean and median LOS distributions for the 11 years in these regions differed significantly. The TFM region continues to maintain, on average, 21% less LOS when compared with SOC. The primary hospitalization and LOS values are summarized in Table 1 and more comprehensively in Table 2.

**Table 1.** Quick Overview of the Statistics for the Individual Health Zones.

Region	Average hospitalization (%)	Mean LOS (days)	Median LOS (days)
South	1.38	0.25	0.14
Calgary	1.00	0.28	0.15
Central	1.44	0.31	0.14
Edmonton	1.50	0.37	0.20
North	1.40	0.24	0.11
TFM	1.10	0.26	0.13
SOC	1.50	0.31	0.15

Normalized hospitalization rates, mean LOS and median LOS are shown in this table. TFM has lower values compared to SOC in all categories. Similarly, Calgary has lower values compared to Edmonton in all categories.

**Table 2.** Two Regions Approach Hospitalization and Mean LOS Values.

		Year										
		’07	’08	’09	’10	’11	’12	’13	’14	’15	’16	’17
TFM	DM	89 881	95 736	103 362	110 590	118 248	125 386	132 686	140 450	148 520	156 545	161 818
	Hosp	1239	1417	1443	1581	1854	1865	1947	2076	2215	2330	2376
	Mean LOS	21	24	23	22	22	23	19	22	20	20	18
SOC	DM	80 428	86 953	93 813	100 595	107 526	113 862	121 617	129 815	137 845	145 640	150 514
	Hosp	968	1022	1091	1148	1274	1329	1361	1420	1441	1592	1692
	Mean LOS	23	23	23	25	24	23	21	21	21	22	20

We used the regional diabetes patient population to normalize hospitalization values.

DM: diabetes mellitus patient population; Hosp: hospitalization; LOS: length of stay.

## Discussion

Managing DFCs is often complex and requires a multidisciplinary approach. Previous studies have demonstrated the effectiveness of multidisciplinary team care in reducing major amputation rates.^12,13^ A systematic review by Musuuza et al found that 94% of studies implementing a TFM approach reported reduced major amputation rates. The review emphasized the importance of addressing glycemic control, vascular disease, infection, and wound care within the multidisciplinary team.^13^ Similarly, in Alberta, Canada, the Calgary zone showed significantly lower rates of major amputations (45%) than similar regions when implementing the TFM approach.^12^ However, in addition to amputations, other DFCs such as cellulitis, pressure ulcerations, and chronic limb-threatening ischemia can also lead to hospital admissions. This study analyzed hospitalization rates for various foot complications related to diabetes and amputations.

The province of Alberta provides an ideal environment for this study, as different clinical regions share the same provincial healthcare system and have similar demographics.^12^ However, only the Calgary region has a dedicated inpatient and outpatient TFM limb preservation program with integrated podiatric surgery services. This study's uniqueness stems from its focus on assessing TFM's influence on hospitalization rates and the duration of patient stays across the expansive and unified backdrop of Alberta. The area is characterized by a unified provincial health care system and consistent demographic profiles, although Calgary is uniquely home to a fully operational TFM center. This distinction allows for a nuanced exploration of TFM's potential benefits within a specific and controlled environment. The podiatry-led multidisciplinary team in Calgary consists of specialists, including podiatric surgeons, vascular surgeons, vascular medicine internists, and infectious disease physicians, who collaborate to provide comprehensive care for DFCs. This program offers robust outpatient management, including endovascular procedures, minor foot amputations, limb preservation surgery, and medical management of vascular risk factors and infections with home-based intravenous therapy. Access to this TFM program is crucial for achieving positive outcomes. Implementing the TFM approach in both inpatient and outpatient settings could reduce major amputation rates, hospitalizations, LOS, and complications associated with DFCs. Additionally, the establishment of a centralized multidisciplinary center in Calgary has significantly enhanced the feasibility and cost-effectiveness of conducting comprehensive research projects related to DFC.^14,15^ This center's multidisciplinary and centralized nature creates an ideal setting for thoroughly and precisely examining the complexities associated with biomarkers and factors related to DFCs.

The study specifically examined hospitalization rates and LOS related to DFCs in Alberta, Canada. The results demonstrated that the TFM group had lower hospitalization rates and shorter LOS per diabetes-related admission than the SOC region. Although both regions showed a decreasing trend in LOS over the 11 years, the TFM region consistently maintained shorter LOS.

One significant advantage of the podiatry-led limb preservation team approach, recognized by professional associations such as the American Podiatric Medical Association and Society for Vascular Surgery, is the efficient management of complex patients through a dedicated limb preservation program with a TFM approach.^16^ Such programs require coordinated care among various specialties and the availability of resources for noninvasive vascular assessment, outpatient endovascular procedures, minor surgeries, diagnostic imaging, wound care, and tissue reconstruction.^17,18^ Developing and establishing such centers ensure timely and appropriate care, leading to standardized practices and reducing disparities within healthcare systems.

A study conducted in Quebec, Canada, revealed the rarity of this model and recognized gaps in best practice recommendations for TFM and integrated care in managing DFUs. They noted the challenges in coordinating and accessing DFU care before and after hospitalization.^19^ Having readily available outpatient multidisciplinary care through limb preservation centers and inpatient care is crucial in addressing these challenges and improving cardiovascular outcomes by identifying vascular risk factors and providing prompt medical management for these complex patients. Stratifying patients into risk groups through screening and implementing treatment protocols, as highlighted by Lavery et al, resulted in a 37.8% decrease in admissions related to foot complications and a 21.7% reduction in inpatient LOS.^20^

Reducing hospitalization rates and LOS has clinical benefits and contributes to significant economic savings. In Quebec, the cost of hospitalization for severe DFUs was estimated to be $70 000 per case. Considering the economic burden in Canada, the cost associated with DFUs was approximately $21 371 per prevalent case.^19,21,22^ In Ontario, DFU-related admissions incurred the highest cost per patient ($22 754) compared to other diabetes-related admissions ($8350).^23^ Therefore, the findings of this study are also promising from a healthcare cost perspective by reducing the financial burden associated with hospitalizations.

### Limitations and Future Directions

Although the regions in this study have similar populations and demographics,^12^ they are not identical. Therefore, other minor confounding variables related to each population likely exist. Given the retrospective nature of this study, not all of these variables can be controlled perfectly.

Given the economic burden related to DFC in Canada, evaluating the economic impact and anticipated reduction in re-admission rates by implementing the TFM approach would be useful to inform these initiatives’ development and resource allocation.

## Conclusion

The findings of this study provide evidence that implementing the TFM can effectively reduce the occurrence of severe DFCs that necessitate hospitalization and decrease the length of hospital stays for those requiring hospitalization. By facilitating collaborative care within a dedicated and comprehensive outpatient center, the TFM enables early identification and management of these complications. These results contribute to the existing body of literature endorsing adopting the TFM approach in healthcare systems, regardless of whether they operate under a single-payer or private insurance model.
